# Sporadic cases of adult measles: a research article

**DOI:** 10.1186/s13104-017-2374-6

**Published:** 2017-01-10

**Authors:** Ranjan Premaratna, Nathasha Luke, Harsha Perera, Mahesh Gunathilake, Pubudu Amarasena, T. G. A. Nilmini Chandrasena

**Affiliations:** 1 Professorial Medical Unit, Colombo North Teaching Hospital, Ragama and Department of Medicine, Faculty of Medicine, University of Kelaniya, Kelaniya, Sri Lanka; 2 Professorial Medical Unit, Colombo North Teaching Hospital, Ragama and Department of Clinical Pharmacology, Faculty of Medicine, University of Kelaniya, Kelaniya, Sri Lanka; 3 Department of Microbiology, Faculty of Medicine, University of Kelaniya, Kelaniya, Sri Lanka; 4 Professorial Medical Unit, Colombo North Teaching Hospital, Ragama, Sri Lanka; 5 Department of Parasitology, Faculty of Medicine, University of Kelaniya, Kelaniya, Sri Lanka

**Keywords:** Measles, Adults, Sri Lanka

## Abstract

**Background:**

Measles caused by a paramyxovirus, characterized by fever, malaise, cough, coryza conjunctivitis, a maculopapular rash is known to result in pneumonia, encephalitis and death. Fatal cases of measles in Sri Lanka are rare after implementation of the National Immunization Programme in 1984. Thereafter 0.1% case fatality rate was observed during October 1999–June 2000 which is a very low figure compared to other regional countries. Immunization guidelines were further revised in 2001, 2011 and in 2012 when additional immunization was recommended to age group 4–21 years; who are likely to have inadequate immunization, in order to achieve elimination of Measles by 2020. However, in 2013–2014, 4690 cases were reported and the majority were children less than 1 year of age. The occurrence in adults is hard to retrieve in published epidemiological reports, however had been 38% (out of 1008 patients) in the 3rd quarter of 2013. During this outbreak 73/101 (72%) reported from the North Central Province of Sri Lanka had been more than 12 years of age with 50% being more than 29 years. 14 Sri lankan adult patients [median age 32 years (range 25–48)] who presented sporadically from June 2014 to March 2016, with confirmed measles infection were enrolled retrospectively after informed consent. Details with regards to their clinical presentation, immunization and other relevant areas were collected using an interviewer administered questionnaire or using patient management records.

**Results:**

The patients presented with high fever, headache, severe body aches, sore throat, dry cough, intense tearing, red eyes and posterior cervical lymphadenopathy over 3–5 days duration. Later they developed discrete maculopapular rash helping the diagnosis. They had a variable degree of leucopenia, lymphocytosis, thrombocytopenia and derangements in the liver functions mimicking any other acute febrile illnesses such as dengue, chikungunya, leptospirosis or Zika virus infection.

**Conclusion:**

At least a 3–5 day delay in the diagnosis was observed (even after the appearance of the rash in some patients), due to non-awareness of its occurrence, unfamiliarity of measles in adults, non-specific nature of the illness and non-availability of rapid diagnostics, risking transmission to the immune-compromised or non-immune staff or patients. Identification of the source of infection in these sporadic adult cases and their virologic surveillance and molecular epidemiology will be important to interrupt the transmission and to achieve the targeted elimination of measles from Sri Lanka by 2020.

## Background

Measles is an acute febrile illness caused by a virus that belongs to the family paramyxovirus in the genus *Morbillivirus*. It is characterized by fever (as high as 105 °F) malaise, cough, coryza, and conjunctivitis, followed by a maculopapular rash [1]. The rash usually appears 14 days after exposure and on the 3rd–5th day of clinical illness which spreads from head to trunk to lower extremities [2]. Measles is usually a mild or a moderately severe illness. However, measles can result in complications such as pneumonia, encephalitis and death. Post infectious encephalitis may occur in approximately one per 1000 reported measles cases. [3, 4]. In Childhood measles, approximately two to three deaths may occur for every 1000 reported measles cases [4].

The annual incidence of measles in Sri Lanka during 1951–1960, 1961–1970 and 1971–1980 varied from about 20 to 47,18 to 38 and 12 to49 per 100,000 population respectively [5]. In 1982, this figure rose to 87/100,000 population. Following this outbreak, measles vaccine was introduced into the Expanded Programme of Immunization (EPI) of Sri Lanka in 1984, with recommendations to be administered at 9 months of age [5]. Morbidity and mortality of measles were reduced remarkably since then and by 1998, the prevalence rate was 0.5/100,000 population [5]. However, from October 1999 to June 2000, 15,204 suspected cases of measles were reported [5]. Among the clinically confirmed cases, 114/100,000 population occurred in less than 9-month-old age group followed by 81/100,000 population in the 15 to 19-year-old age group. Nearly 54% of the cases were above 15-year-old age suggesting a gradual shift of the disease to the older age groups [5]. During this outbreak, vaccination history was available in 3728 clinically confirmed cases. Of them 60% had not been vaccinated; 10% in the 5 to 9 year-old age group, and 38% in the 10 to 14-year-old age groups and of those who were above 15 years of age,more than 80% had not been vaccinated against measles [5]. There were 5 deaths, giving a case-fatality rate of 0.1% [5]. The ages of those who died were 9 months and 4, 5, 17, and 24 years. Therefore, it was suggested that despite high immunization coverage, additional susceptible persons could be expected to join this non-immune cohort because the vaccine is only 85% effective [3, 4, 6]. Therefore, a second dose of measles vaccine was introduced to all children at the age of 3 years since 2001 in combination with rubella vaccine [MR (Measles and Rubella vaccine)]. In 2011 however MMR [Mumps, Measles and Rubella] vaccine was introduced at the age of 1 year and 3 years replacing measles vaccine at 9 months and MR vaccine at 3 years [7]. Furthermore, in March 2012, an additional immunization programme was instituted to cover the age group 4–21 years with view to cover all those who are likely to have had no or single dose immunization [7]. This is because, the “Revised Measles, Rubella, Congenital rubella syndrome elimination targets: September 2015” intends to eliminate measles from Sri Lanka by the year 2020 [8]. After 12 years of introduction of MR vaccine, from 2nd quarter of 2013 to 1st quarter of 2014, an island wide outbreak of nearly 4690 cases were reported from the country [9]. Of which, nearly 26% had been in the less than 1 year of age and nearly 38% had been in the age >12 years [10]. During this outbreak, 101 measles suspects were reported from Anuradhapura Teaching Hospitalin North Central Province of Sri Lanka, and 73/101 (72%) of them had been more than 12 years of age. Of the 73, nearly half were more than the age 29 years. Authors suggest change in the immunization schedule in 2012 as triggering factor for this outbreak [11].

During the period, June 2014 to March 2016, 110 patients with measles were reported from the Colombo North Teaching Hospital (CNTH), Ragama, Sri Lanka and included 68 patients less than 12 years of age and 42 above 12 years of age (unpublished hospital data). Of them, we report clinical and demographic data of 12 adults with measles who presented to the Professorial Medical Unit of CNTH and 2 patients who were admitted to a Private hospital in Ragama in order to highlight several considerations that was thought important when dealing with adult patients with measles infection.

## Methodology

A retrospective study was carried out recruiting 14 adult patients with confirmed measles infection who were managed at the Professorial Medical Unit, Colombo North Teaching Hospital, Ragama, Sri Lanka during June 2014 to March 2016. Demographic and clinical data were retrieved retrospectively using a predesigned questionnaire with the help of ward records. Some other relevant data were later obtained by interviewing patients during their clinic visits and by telephone conversations.

## Results

**Fig. 1:**
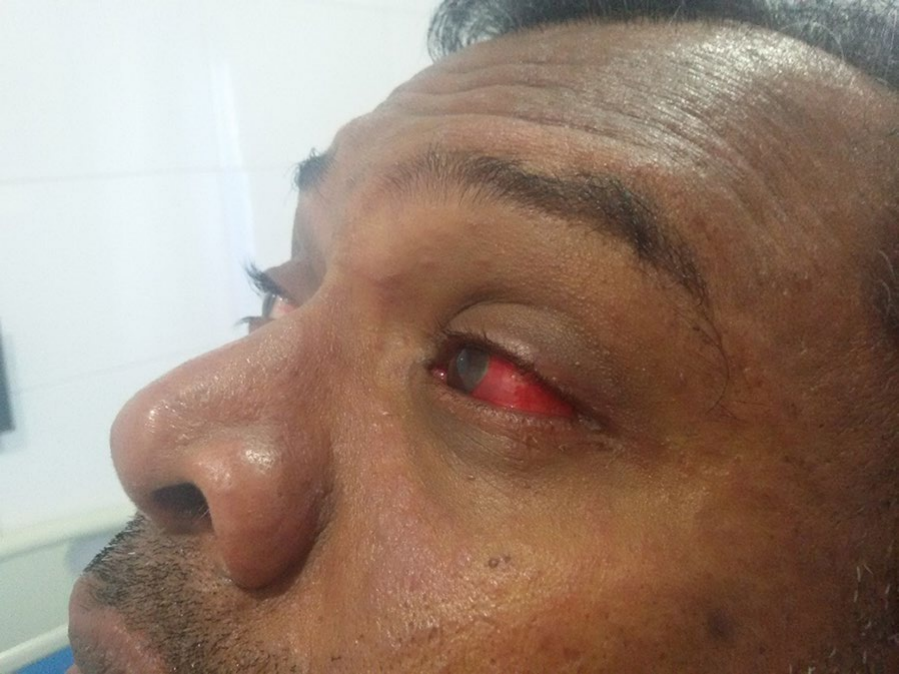
*Red* eyes in a patient with measles

**Fig. 2:**
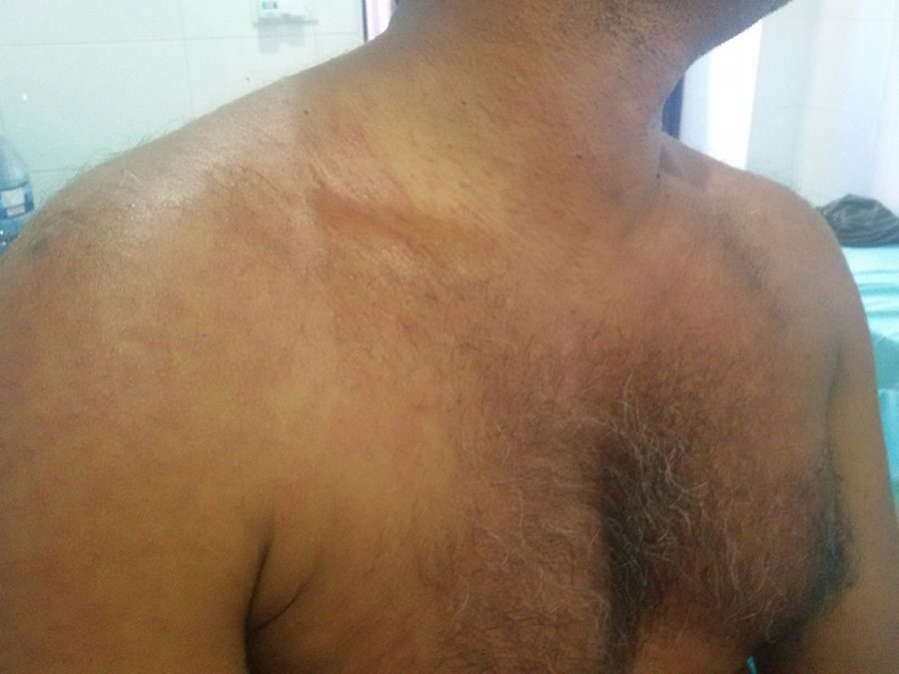
Maculopapular rash of measles

Fourteen adult patients;8 males,with median age 32(range 25–48) years,presented with high fever, headache, severe body aches, a severe sore throat and a dry cough for 2–3 days duration. They complained of severe sore eyes around the 4–5th day associated with intense tearing. Examination revealed variable degree of posterior cervical lymphadenopathy and in 6 of them, typical Koplik’s spots were noted in the buccal mucosa on the 3–4th day of illness. The eyes were injected around the 4th day and progressed to develop red eyes over the next few days (Fig. 1). Five of them developed sub-conjunctival haemorrhages. The patients with severe conjunctivitis had photophobia and intense tearing requiring treatment by ophthalmologists. Around the 5th day of clinical illness, they developed a discrete maculopapular rash starting in the forehead and the face which spread to involve the upper chest, back and the limbs including palms and soles by the 7th day (Fig. 2). Almost all patients developed a dry cough on the 3rd day and 8 developed severe intractable cough and wheezing with the chest examination revealing wide spread coarse crackles and polyphonic rhonchi. In investigations on 3rd–4th day; the white cell count ranged 2.2–6.2 × 10^3^/dL with neutrophils ranging 34–53% and lymphocytes 35–55%. The lowest platelets ranged 96–152 × 10^9^/L. The C-reactive protein levels (CRP) ranged from 14 to 56 mg/dl. Almost all of these patients had derangements in the liver functions with ALT (Alanine aminotransferase) median (range) 246iu/L (128–367) and AST (Aspartate aminotransferase)178iu/L (96–236) with normal serum bilirubin and alkaline phosphatase levels. Around the 5–7th day, eight patients had coarse crackles wide spread over the lung fields, rises in the white cell counts (9.3–11.5 × 10^3^/dL; N 65–78%), high CRP levels (24–110 mg/dL) and chest radiographs showing bilateral patchy opacifications (2–5 mm) suggestive of varying degrees of lower respiratory tract involvement and required management with intravenous cephalosporins (cefuroxime or cefotaxime). Altogether, the illness lasted up to about 10 days and had high fever spikes ranging from 102 to 104 °F for about 6–7 days in a majority of cases. The fever regressed with the fading of the rash and was followed by peeling of the facial skin and the palms and soles. All 14 patients were positive for measles IgM antibodies confirming the diagnosis. They were negative for dengue NS1 Ag. On further questioning none of these patients knew whether they had measles immunization as a child nor had immunization records available with them. Furthermore, they were not aware of recent recommendation of immunization for those who were above 25 years of age. When questioned whether they would have obliged to obtain such immunization, they raised doubts as to whether such immunization is really necessary at their age. While only 10 of themhad heard of measles, 8 thought it was an illness in the childhood and adults had nothing to do with it. Furthermore, two patients had regular foreign travel to regional countries for business or occupation related purposes, one had travelled to India on Buddhist pilgrimage several occasions over the last four years. None of the others have travelled outside the country. While one patient who arrived for follow up visit had become aware that the receptionist in his private company had been ill with a similar illness about two days prior to his illness, none of the others were aware of such illness among their work mates or among other associates. However they were travelling in public transport very often for daily needs. The last patient to be included in the cohort was a sister of a final year medical student who had been doing the paediatric clinical appointment at the time she developed the illness.

## Discussion

Eradication effort has to permanently eliminate a pathogen everywhere in the world thereby removing the risk of reintroduction and re-establishment. Elimination, on the other hand, focuses on reduction to zero incidence of a certain pathogen in a given area, with active measures to prevent pathogen re-establishment from other areas after elimination. During an elimination process, once an infection is driven to very low levels, the ecology of pathogens may change requiring different surveillance and control strategies [12]. Susceptible build-up, waning of immunity, increase in the age of infection, non-compliance of individuals with control measures, pathogen change and emergence of resistance as a result of intensified efforts all become increasingly important during the final stages of such programmes [12]. Therefore, in order to achieve targets for elimination of vaccine preventable highly contagious illnesses such as measles, in addition to the maintenance of vaccination integrity, all other likely contributory factors for the illness in the community needs to be timely identified and intervened.

CNTH is the only tertiary care institution in the Gampaha district of the Western Province of Sri Lanka. Although it does not represent the status of hospitals in the whole of Sri Lanka, Western Province or the Gampaha district, the two hospitals included here are likely to represent the basic infrastructure facilities that are available in most of the government hospitals and the private sector hospitals. Firstly, the adult patients who presented to both hospitals had at least 3–4 days of admission until the diagnosis was made. The patients who were admitted to CNTH were kept in a busy medical unit, among most other patients with either acute or chronic ill health. The reasons for the delay in the diagnosis included non-awareness of its occurrence, unfamiliarity of clinical illness among adults and non-specific nature of the clinical illness that mimicked any other common acute tropical febrile illness.

Since all these patients were above 25 years of age it is likely that they had no or partial immunization against measles by natural mechanisms or through National Immunization programme. During the acute phase of illness (first 4 days) all these patients presented with an illness that mimicked acute dengue or any other common acute febrile illnesses in the tropical setting. Although they were negative by NS1 antigen they were kept under close monitoring as almost all of them had reducing or low platelet counts and derangement in hepatic enzymes. Although the development of the facial rash together with red tearing eyes suggested the possibility of measles, its diagnosis was delayed at least in the first few patients due to unfamiliarity of the illness in adults. It mimicked chikungunya fever in some cases due to severe arthralgia and the non-specific nature of the rash and its pattern of involvement [13]. Furthermore, a similar clinical illness seems to occur in Zika virus infection [14] although it is yet to be documented in Sri Lanka.

Almost all of these patients were managed in a busy clinical setting and amongst other patients with acute or chronic illnesses such as diabetes mellitus, chronic liver disease or chronic kidney disease where immune deficiency is known to occur. In multi-specialty tertiary care centers there is a possibility of transmission to immune-compromised and pregnant patients as well as to non-immune staff who may in turn care for high-risk patients [15]. Although we were not made aware of any occurrence of measles among patients or the ward staff who had close contact during the management of these patients, influx of patients with measles to busy multidisciplinary tertiary care centers during community outbreaks of measles have resulted in nosocomial outbreaks [16, 17].

Early diagnosis of measles and isolation of such patients would be the most ideal strategy in order to prevent spread and occurrence of spread to other non-immune patients or health care workers. However currently there are no early diagnostic facilities available for measles and there are no isolation facilities available in CNTH or most other major hospitals in Sri Lanka. Furthermore, it is very unlikely that such facilities would be realistic in most hospitalsof developing countries such as Sri Lanka. It is well known that acute febrile illnesses that occur in the tropics such as measles, dengue fever, typhoid fever, leptospirosis, and severe acute respiratory syndrome (SARS) can be confused with each other [18]. At presentation, these febrile illnesses share similar clinical features, including headache, myalgia, and rash. In the case of dengue fever clinical features of dengue haemorrhagic fever, such as bleeding and plasma leakage, are seen after the initial febrile phase is subsiding, typically after the third or fourth day of fever. Similarly rash of measles although non-specific, appears after the 4th day of the illness. Therefore illnesses with similar characteristics, such as dengue, leptospirosis, measles, etc. have been found difficult to discriminate on the basis of any clinical algorithm alone [18]. Therefore, the most important strategy to diagnose measles at a very early stage would be to develop molecular/antigen based rapid diagnostics with high sensitivity and specificity. However, it is very unlikely that such diagnostics would be developed and made available, cost effective or realistic for illnesses such as measles due to its low incidence and low mortality figures.

Although the most likely reason for measles infections in these adult patients would be the on-going low level measles infection in the community, we feel that it is important to consider other likely source of the measles virus especially in the adults. Such sources could be related to acquisition of the virus through foreign travel although none of these patients or their close contacts had a history of foreign travel closer to their being ill. This is because such infection is likely to introduce non-native genotypes of the virus resulting in severe outbreaks [19]. Furthermore potential for introduction of new virus-genotypes should be kept in mind asworld travel is rapidly expanding for many purposes such as for education, employment and tourism. Travel has been shown to cause importation of infections such as SARS, chikungunya, dengue and Zika virus to many parts of the world resulting in outbreaks or establishment of these infections in various geographical regions [20, 21]. Therefore, there is always a risk of transmission of illnesses like measles between countries.

The combination of molecular epidemiology and standard case classification and reporting seems to be very sensitive means to describe the transmission pathways of measles. Virologic surveillance in order to monitor the viral genotypes in a particular country or region over time seems very important in order to interrupt the transmission of endemic measles [22]. In order to address these issues, the Epidemiology Unit of Sri Lanka has implemented due measures to obtain comprehensive data collection from patients with measles including obtaining blood samples for genetic isolations [23]. However, it was noted that such operations have either been unaware of or slowed down due to sporadic nature of measles among the adult patients (unpublished data). Therefore, intensified public awareness, periodic reminding of medical institutions and implementation of proper monitoring systems may be warranted in order to achieve current goals towards elimination of measles by the year 2020.

## Conclusion

In conclusion, clinicians who deal with adult patients should be made aware of the possibility of measles infection among acute febrile illness and be reminded of the symptomatology of measles in adults. Furthermore, it would be essential to understand the community implications of these sporadic cases of adult measles towards the ongoing efforts on eradication of measles both locally and globally. While availability of early diagnostic facilities would help in management of patients, isolation of the virus and genotyping are likely to play an important role in implementing effective immunization and help achieving the targeted elimination of measles by the year 2020.

## Abbreviations

SARS: severe acute respiratory syndrome
EPI: Expanded Programme of Immunization
MMR vaccine: mumps, measles and rubella vaccine
MR vaccine: measles and rubella vaccine
ALT: alanine aminotransferase
AST: aspartate aminotransferase
CRP: C- reactive protein
CNTH: Colombo North Teaching Hospital
